# A Dutch panel study on the relation between structure of everyday life, daily hassles, and alcohol consumption

**DOI:** 10.1186/1471-2458-12-1068

**Published:** 2012-12-11

**Authors:** Rik Crutzen, Ronald A Knibbe

**Affiliations:** 1 Department of Health Promotion, Maastricht University/CAPHRI, P.O. Box 616, 6200 MD, Maastricht, The Netherlands

**Keywords:** Structure of everyday life, Daily hassles, Alcohol consumption, Panel study, General population

## Abstract

**Background:**

A widely held assumption within the general public is that one way in which people cope with their daily hassles is by drinking alcohol. Although the idea of drinking to compensate for daily hassles is intuit, empirical evidence is actually rather scarce. This study aimed to test whether structure of everyday life results in more daily hassles and has a protective effect regarding alcohol consumption (as predicted by classic role theory) or – in case the relation between daily hassles and alcohol consumption is positive (as predicted by tension reduction theories) – daily hassles would decrease the protective effect of having a more structured everyday life.

**Methods:**

A general population panel study (*N* = 2,440; 47% women; age: M = 52 years, SD = 17), measuring structure of everyday life and daily hassles (T1; 90% response rate) as well as alcohol consumption (T2; 85% response rate).

**Results:**

In line with classic role theory – structure of everyday life was positively associated with daily hassles and had a negative effect on alcohol consumption. Daily hassles was not associated with alcohol consumption.

**Conclusions:**

Daily hassles did not mediate the relationship between structure of everyday life and alcohol consumption.

## Background

A widely held assumption within the general public is that one way in which people cope with their daily hassles is by drinking alcohol, also denoted as “irrational coping” [1]. Participants in a qualitative study, for example, spoke of “relaxing with alcohol after a hard day’s work” [2]. Assuming that daily hassles indicate stress [3], this idea is in line with tension reduction theories stating that drinking alcohol could reduce stress-related tension [4]. A recent study showed that daily hassles predict increased alcohol consumption the following day among heavy drinkers [5]. Another study using daily diary methodology, however, found only weak evidence of perceived stress mediating the associations between daily experiences and alcohol consumption [6]. Furthermore, a 2-year longitudinal study among a 27-29-year-old sample revealed no relationship between changes in daily hassles and alcohol consumption [7]. Although the idea of drinking to compensate for daily hassles is intuit, empirical evidence is actually rather scarce.

There is another theory that at first sight assumes almost the opposite: the busier everyday life is, the less opportunity to drink. The theory which is most articulate in stressing the relevance of structure of everyday life for alcohol consumption is the classic role theory [8,9]. According to this theory, the more everyday life is structured, the less chance there is on heavy or excessive drinking, because roles that structure daily life might also lead to daily hassles that reduce opportunity to drink. The roles that most strongly determine how structured everyday life is are the roles of living with a partner, taking care of children and having paid work. These roles limit the opportunity of some to participate in drinking situations and will increase social control (from partners, family, and colleagues) on any drinking that would interfere with the adequate performance of these roles. There is good support for this theory [9-12]. A pioneer study from 1987 already confirmed the idea that people with a less structured everyday life are inclined to intensify their alcohol consumption [9]. More recently, in a study in 10 western industrialised countries, it appeared that those who had all three roles were least likely to drink heavily or engage in risky single occasion drinking, thus supporting the assumptions of classic role theory [10]. This role theory leads one to expect that daily hassles may mediate the negative relationship between structure of everyday life and alcohol consumption.

**Figure 1:**
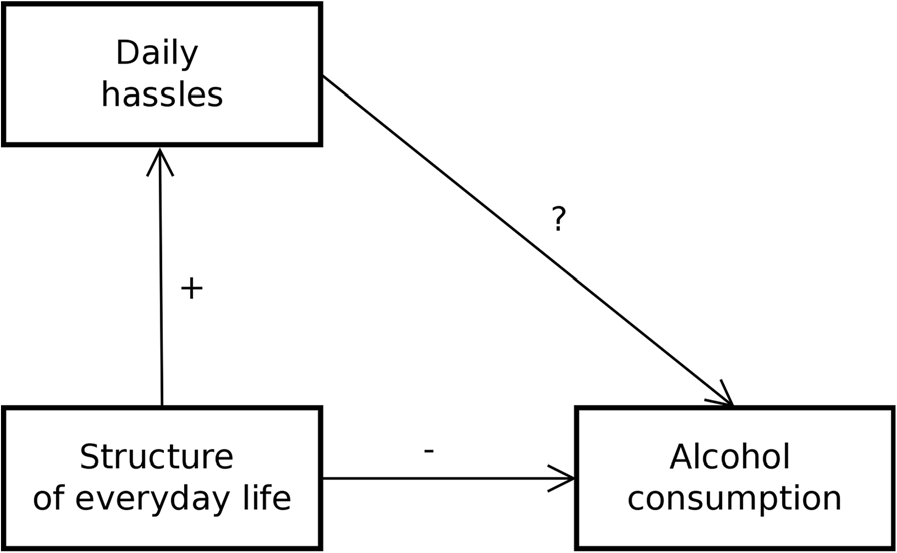
Conceptual model.

Figure 1 shows the conceptual model that was tested within this study and illustrates (1) that structure of everyday life is hypothesized to result in more daily hassles and has a protective effect regarding alcohol consumption and (2) that the question to be answered is whether daily hassles mediate the relationship between structure of everyday life and alcohol consumption.

## Methods

Data were collected through the LISS panel (Longitudinal Internet Studies for the Social sciences; http://www.lissdata.nl) [13]. The reference population for the LISS panel is the Dutch speaking adult population of 18 years and older permanently residing in the Netherlands. In co-operation with Statistics Netherlands addresses were drawn from the nationwide address frame including individuals who do not have Internet access. These were provided equipment to access the Internet via a broadband connection to ensure that our sample was not limited to people with Internet access. Those with small band Internet access were provided with broadband. There was ethics approval for the umbrella project, which was conducted by an external party (CentERdata; http://www.centerdata.nl/en). Only data regarding structure of everyday life, daily hassles, and alcohol consumption was used for the study at hand. Relevant ethical safeguards were met with regard to the participant confidentiality and consent.

### Participants

A random sample of 3,192 potential respondents was drawn from those panel members that indicated in November 2010 that they drink alcohol (i.e., “did you have a drink containing alcohol over the last 12 months?”). These were invited for the first measurement (T1) regarding structure of everyday life and daily hassles in January 2011. Age, gender, and educational level (low = primary school/intermediate secondary education; intermediate = higher secondary education/preparatory university education/intermediate vocational education; high = higher vocational education/university) were known for all panel members. Of those invited, 2,861 responded (90% response rate) and were invited for the second measurement (T2) regarding alcohol consumption in April 2011. Of those invited for the second measurement, 2,440 responded (85% response rate). There was no selective dropout regarding gender (χ^2^(1, *N* = 2861) = .23, *p* = .64) and educational level (χ^2^(2, *N* = 2861) = .04, *p* = .98), but those that dropped out were younger (45 vs. 52 years, *t*(2859) = 8.98, *p* < .001).

### Measures

#### Structure of everyday life

Partnership (either being married or cohabiting), parenthood (i.e., the existence of children in the household), and paid labour (either being employed or self-employed) were assessed by single items and used as indicators for structure of everyday life. All these indicators were dichotomized and the sum of these was used as an index (score 0–3) for the structure of everyday life (in line with previous studies) [9,10,14]. We also report when the direction of effect of any of the separate indicators for structure of everyday life differed from the direction of effect of the index score.

#### Daily hassles

Stress related to daily hassles was assessed by the Survey of Recent Life Experiences [15]. Participants had to indicate for eight time pressure related experiences (e.g., “not enough leisure time”; “too many things to do at once”) how much this was part of their life in the past three months. Answer categories were ‘not at all part of my life’, ‘only slightly part of my life’, ‘distinctly part of my life’, and ‘very much part of my life’ (possible range mean score 1–4, Cronbach’s α = .81).

#### Alcohol consumption

Two measures of alcohol consumption, reflecting respectively quantity and frequency of drinking were assessed: (1) the number of drinks on the heaviest drinking day during the past week and (2) the number of drinking days in the past week (0–7). To increase validity, respondents were asked to report each type of beverage separately (e.g., strong beer, extra strong beer, strong drinks/liqueur, sherry/martini, wine and premixes). Also the type of serving was assessed (e.g., a regular glass, a pint, a small or large can, a small or large bottle). These drinks were converted into standard drinks based on the alcohol content in most on-premise locations in the Netherlands: a standard drink content of 10 grams alcohol [16].

### Analyses

Using Mplus 5 (Muthén & Muthén, Los Angeles, CA), a structural equation model was constructed to test the conceptual model. Maximum likelihood parameter estimates with standard errors that are robust to non-normality and non-independence of observations were used, since both measures of alcohol consumption were non-normally distributed. Daily hassles and alcohol consumption were included in the model as latent constructs. Alcohol consumption was regressed on structure of everyday life and daily hassles. Daily hassles was regressed on structure of everyday life. The model was age-adjusted to account for selective dropout and the strong correlation with position roles (*r* = −.46). The model was also education-adjusted because those higher educated are known to report more daily hassles in comparison with those lower educated [17].

## Results

**Figure 2:**
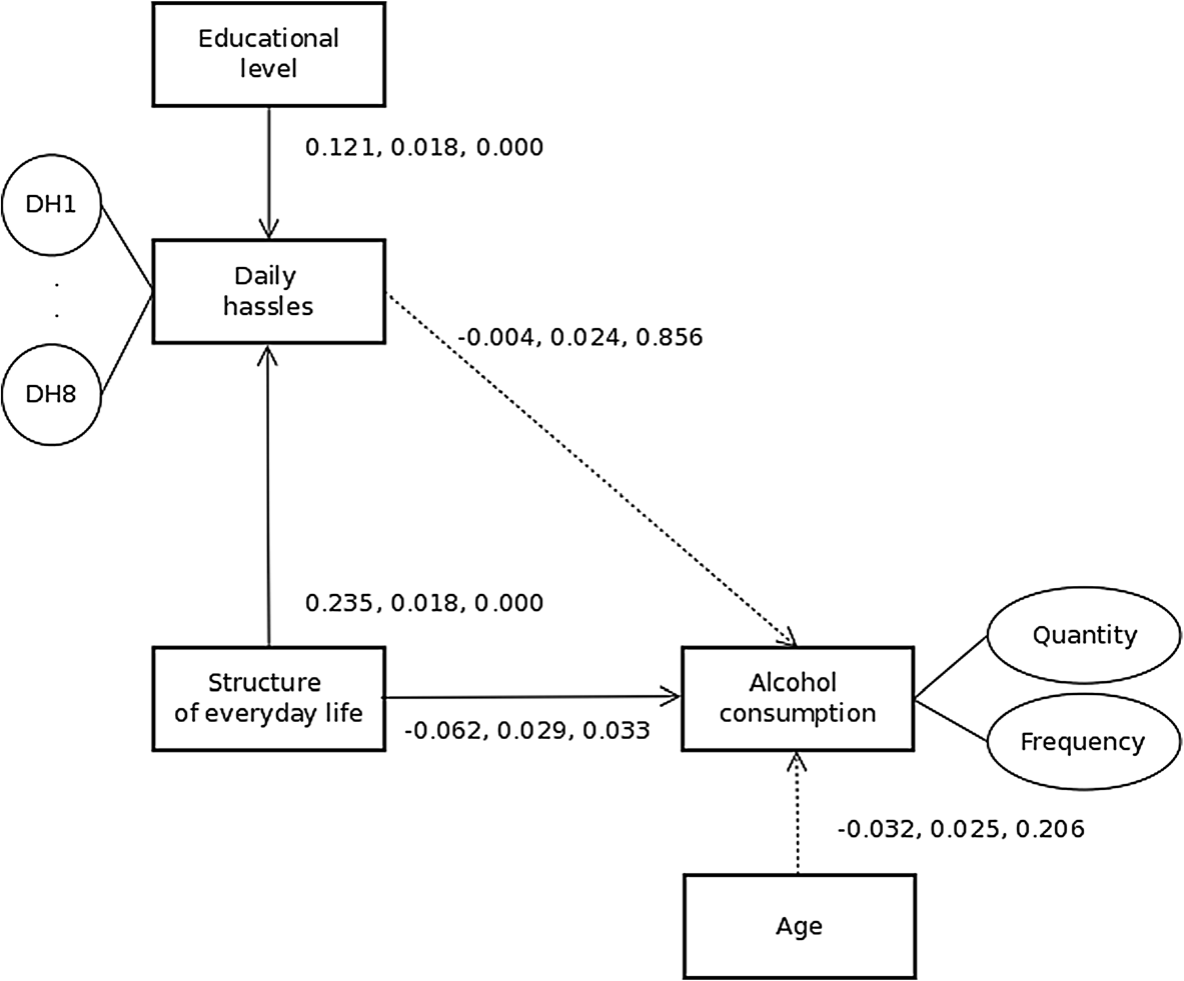
**Structural equation model for relationships between structure of everyday life, daily hassles, and alcohol consumption.** Note: Numbers next to paths indicate respectively estimates, standard errors and p-values. Dotted arrows indicate non-significant paths. Circles indicate items that load on latent constructs.

Table 1 provides an overview of the sample characteristics and Figure 2 shows the final model that results from the analysis. The relation between structure of everyday life and daily hassles is as expected: the more structured everyday life is, the more daily hassles are reported. The relation between structure of everyday life and alcohol consumption is also as expected: the more structured everyday life is, the lower the alcohol consumption. However, considering the lack of a relation between daily hassles and alcohol consumption, there is no support at all for drinking because one is too busy and neither for daily hassles mediating the relation between structure of everyday life and drinking.

**Table 1:** **Sample characteristics (*N*** **= 2440)**

**Variable**		**Descriptive statistics**
Age		M = 52 (SD = 17)
		Range 18–85
Gender		47% women
Educational level	Low	34%
	Intermediate	32%
	High	34%
Structure of everyday life	Partnership	76%
	Parenthood	36%
	Paid labour	51%
Daily hassles (1–4)		M = 1.6 (SD = 0.5)
Alcohol consumption	Drinks on heaviest drinking day	M = 3.6 (SD = 4.0)
	Drinking days (0–7)	M = 3.5 (SD = 2.4)

## Discussion

The results of this study are straightforward: Individuals with more structured lives tend to drink less. Daily hassles are not associated with alcohol consumption, neither positive nor negative. The lack of an association between daily hassles and alcohol consumption in this study confirms that tension reduction theories are overly broad [18]. How could this lack of association between daily hassles and alcohol consumption be explained?

First, drinking alcohol is not the only behaviour triggered by daily hassles. The association between daily hassles and alcohol consumption could be stronger among people who believe that drinking alcohol is the only way to reduce tension. One study found no differences between alcoholics and non-alcoholics who were asked to assess level of stress associated with various scenarios combining life-change events or a social situation and an emotional state. However, despite the similarity in perception of stressfulness, non-alcoholics reported little stimulus to drink, whereas alcoholics perceived the more stressful scenarios as stimulating an urge to drink [19]. Participants in the study at hand are, in contrast to alcoholics, likely to differ in terms of behaviours triggered by daily hassles. In other words, drinking alcohol might be behaviour less likely to be triggered by daily hassles among a general population sample in comparison with a sample consisting of alcoholics. This decreases the likelihood of finding an association between daily hassles and alcohol consumption in a general population study.

Second, daily hassles are not the only reason to drink. A study among middle-aged women revealed that problem-focused coping moderated the relationship between stress and alcohol [20]. Furthermore, a positive relationship has been found between the occurrence of negative life-events and alcohol consumption in people scoring high on emotion coping, and a negative one among people scoring low on emotion coping [21]. It is possible that daily hassles are only positively associated with alcohol consumption if people drink mainly for coping motives. Coping motives are one of the four motives according to the motivational model of alcohol use and concern drinking to avoid negative emotions [22]. If people frequently have this motive to drink, then it might be that for these people daily hassles mediate the relation between structure of everyday life and drinking. A previous study showed that coping motives are less frequently indicated in comparison with enhancement motives (i.e., to enhance positive mood) and social motives (i.e., to obtain social rewards) [23]. Therefore, it is unfounded to presume that daily hassles mediate the relation between structure of everyday life and drinking in general, because this might depend on the relative frequency of coping motives. Although future research needs to shed light on this hypothesis, it is in line with a previous suggestion that “a ‘stress and coping’ framework should be incorporated in role analysis in order to increase its explanatory power” [24].

It needs to be stressed that participants scored mostly at the lower-end of the daily hassles-scale with limited variation (i.e., a small standard deviation), but given the sample, this is representative of the variation regarding daily hassles in the general population, meaning that there is no such association in the general population. A limitation of this study, however, is the difference in time frame regarding measurements of daily hassles (i.e., three months) and alcohol consumption (i.e., one week). Post-hoc analysis using cross-sectional data regarding alcohol consumption, however, revealed a similar pattern regarding the effects of daily hassles on alcohol consumption (−0.004 longitudinal vs. 0.009 cross-sectional, both non-significant) and structure of everyday life on alcohol consumption (−0.062 longitudinal vs. −0.066 cross-sectional, both significant), confirming the current findings.

Another limitation is the crude measures of structure of everyday life and alcohol consumption. Taking into account the number of children (as opposed to the existence of children in the household) or being either part-time of full-time employed (as opposed to having paid labour) might provide more insight into the relationship between structure of everyday life and alcohol consumption. The use of dichotomous indicators, however, is common when using the role theory as theoretical framework for explaining alcohol consumption [9,10,14]. Future research might also look at the strength of the association between various roles and daily hassles. Even if the direction of association is likely to be the same, there might be differences in terms of effect size. With regard to alcohol consumption, it is furthermore worthwhile to study not only the frequency and quantity of drinking, but also heavy episodic drinking and the specific context in which the drinking takes place. For example, the location (e.g, home vs. licensed establishments) [25] or the number of locations in the course of an evening [26]. These contextual factors could contribute to further explaining the relationship between structure of everyday life and alcohol consumption, e.g., the parental role may be associated with variation in where people drink, and where people drink may be associated with variation in heavy drinking. A previous study found that for women, parenthood is related to a reduction in heavy drinking associated with a reduction of drinking occasions that occur at bars. For men, parenthood is related to a reduction in heavy drinking partly because fathers more often drink at friends’ homes and the proportion of drinking occasions that occur at bars is smaller among fathers than non-fathers [27].

Finally, although the reference population for the panel used in this study is the Dutch speaking adult population permanently residing in the Netherlands, the final sample is not necessarily representative of this population because there was selective dropout regarding age. Despite the final sample being relatively old, there is still good variation regarding participants’ age, gender, and education level to warrant generalizability of the findings.

## Conclusion

Daily hassles are not associated with alcohol consumption, neither positive nor negative.

## Competing interests

Both authors declare that they have no competing interests.

## Authors’ contributions

RC initiated the study, analyzed the data and wrote the first draft of the manuscript, RK revised the manuscript critically for important intellectual content. Both authors read and approved the final manuscript.

## Pre-publication history

The pre-publication history for this paper can be accessed here:

http://www.biomedcentral.com/1471-2458/12/1068/prepub
